# Latissimus dorsi tendon transfers: a historical journey

**DOI:** 10.1051/sicotj/2021009

**Published:** 2021-03-08

**Authors:** Ismail Turkmen, Emre Koraman, Oguz Poyanlı

**Affiliations:** 1 Associate Professor, Department of Orthopaedics and Traumatology, Goztepe City Hospital Egitim mh Dr. Erkin cd 34722 Kadikoy Istanbul Turkey; 2 MD, Department of Orthopaedics and Traumatology, Goztepe City Hospital 34722 Kadikoy Istanbul Turkey; 3 Professor, Department of Orthopaedics and Traumatology, Goztepe City Hospital 34722 Kadikoy Istanbul Turkey

**Keywords:** Upper extremity, Tendon transfers, Latissimus dorsi, Rotator cuff

## Abstract

Several latissimus dorsi tendon transfer techniques for shoulder problems have been previously described and developed. These techniques involve the transfer in obstetric palsy, transfer in irreparable posterosuperior rotator cuff tears and subscapularis tears, and transfer in reverse shoulder arthroplasty. We detail the differences in planning and surgery and the need for different approaches. This historical and technical description provided in this study will benefit surgeons wishing to use the procedure.

## Introduction

Rotator cuff (RC) tears are a common shoulder problem that causes pain and limitation of movement and may result in arthropathy [1]. In acute and nonmassive tears, RC repair methods are the first-line surgical treatment [2]. Various surgical options are available for massive rotator cuff tears, including tendon transfers, shoulder arthroplasty, and partial repair [3–6]. Among these options, tendon transfer is a surgical procedure aimed at providing near-complete function in massive and irreparable rotator cuff tears [7]. Among tendon transfers, latissimus dorsi (LD) transfer is a method that has been combined and modified with various surgical techniques over the years [7]. The resurgence in the use of the technique, modifications, and location of the transfer has prompted a review of this important procedure.

### Anatomy and historical perspective

**Figure 1:**
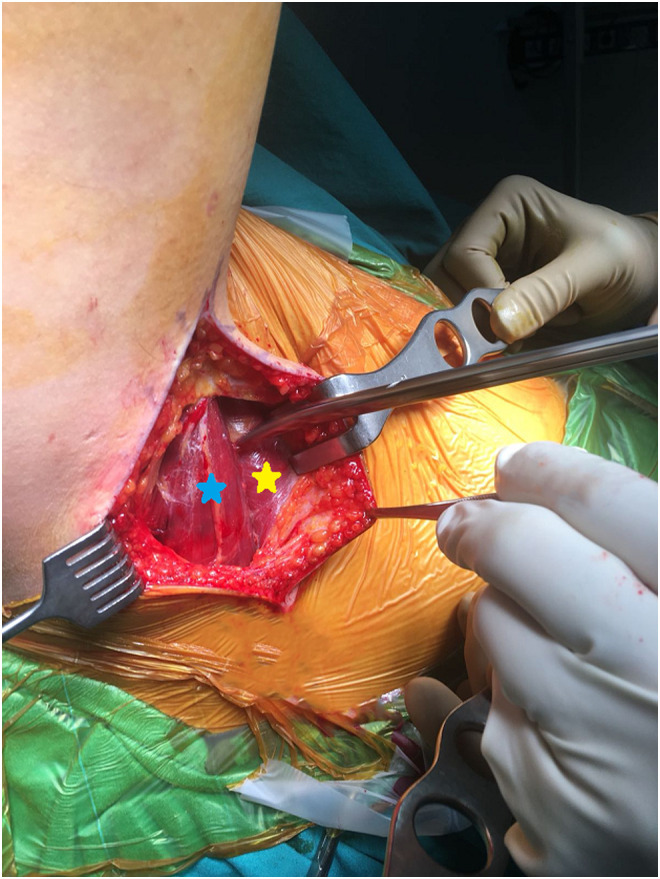
Latissimus dorsi muscle (*yellow asterisk*) and its relationship with teres major (*blue asterisk*). LD muscle is more anterior compared to teres major.

The LD originates from the T7–L5 vertebrae spinous process, thoracolumbar fascia, iliac crest, inferior margin of the 3rd and 4th costae, and inferior margin of the scapulae [8]. The LD inserts into the base of the intertubercular groove locate more anterior compared to teres major (Figure 1) in the humerus and is fed from the thoracodorsal branches of the subscapular artery and innervated by the thoracodorsal nerve, consisting of the roots C6, C7, and C8 [2]. The LD is the widest muscle of the body and plays a role in adduction and extension in the humerus and internal rotation in the adduction [8]. Indirectly, it retracts and depresses the scapula. Deltoid and trapezius muscles are antagonists of LD [2, 8]. In some studies, it has been stated that the medial and lateral parts of the LD muscle have different functions and this difference is due to the two main branches of the thoracodorsal nerve [9, 10]. The LD plays a role in the stability of the glenohumeral joint [2] by helping to create a stable scapular platform in response to glenohumeral joint motion along with other shoulder girdle muscles [2].

The basic working principle of tendon transfer in massive RC tears is to separate a tendon of a muscle near the humerus head and fix it to the RC insertion [5]. Due to the previously mentioned anatomical features, the LD tendon can help close the defect in massive RC tears as a wide, vascularized tendon [11]. Functionally, the LD allows for deltoid activity on the one hand, and on the other hand, restores the shoulder’s external rotation [11]. LD transfer has been a frequently used tendon transfer technique in posterosuperior RC tears for many years [7]. The success of this technique in reducing pain in the patient and in recovering the RC to a functional state has been demonstrated in previous studies [12, 13]. The LD was first used by surgeons in the 1800s as a musculocutaneous flap after breast cancer surgery [14, 15]. The first time that an article was published on the technique was by D’Este [14]. The starting point of its use in the shoulder and upper extremity surgery is the surgical treatment of obstetric palsy [16]. The techniques used in today’s orthopedic surgery evolved by modifying the LD tendon transfer technique.

### Transfer in obstetric palsy

**Figure 2:**
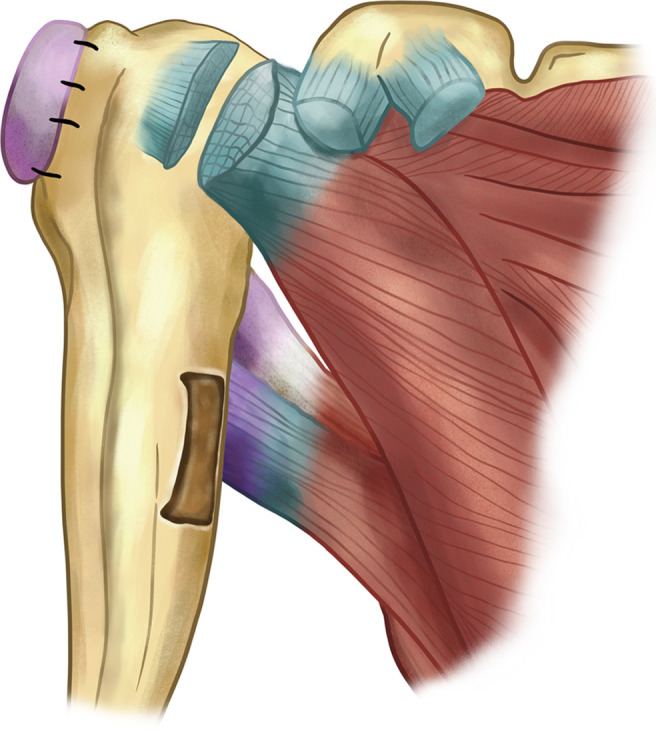
The L’Episcopo procedure. The contracted anterior tissues, such as subscapularis tendon, are released using Sever’s technique. Teres major and LD are detached from their insertions as an osteoperiosteal flap. Combined transfer of the tendons of LD and teres major to the posterolateral aspect of humerus aims to achieve external rotation in obstetric plexus paralysis.

In 1934, L’Episcopo described the transfer of latissimus dorsi and teres major muscles to restore external rotation in patients with obstetric plexus palsy [16]. Referring to the Lange osteotomy Sever’s relaxation procedures and Kleinberg osteotomies, L’Episcopo argued that the applied osteotomy and relaxation techniques were only aimed at correcting deformity but did not eliminate the problem of muscle imbalance [2]. The L’Episcopo procedure, based on the philosophy of correction of muscle imbalance, was first implemented in 1931 for residual or neglected older children [2]. In patients with obstetric plexus palsy where the technique was applied, there were also internal rotation deformity and contractures in the internal rotator and adductor muscles [16]. These pathologies caused situations that negatively affected their lives [2]. According to the new concept supported by L’Episcopo in those years, in addition to releasing the contracted soft tissues, it has been argued that deformity corrective forces should be restored against deformity-forming forces [17]. The results will be better if the deformity-forming effects of the internal rotators are countered with one or more tendons [17]. In the first surgery performed in 1931, contracted tissues were first released with an anterior incision, similar to that of Sever’s technique [17]. With a second posterior incision, the teres major tendon was removed from its insertion and was sutured in the form of an osteo-periosteal flap to cover the short head of the triceps [17]. The tendon thus surrounds the humerus not from the posterolateral but from the posteromedial position [18] (Figure 2). After surgery, patients were placed in a cast in arm abduction and external rotation, forearm supination, and this cast was removed after 3 months [18]. Only teres major muscle was used in the first surgery [16]. The idea of using a new route to the internal rotator muscle as an external rotator muscle is attributed to L’Episcopo [18]. This technique has been modified over the years to be applied without subscapularis disinsertion or release. Özben et al. applied pectoralis major tendon Z-plasty in addition to latissimus dorsi transfer in the treatment of obstetric palsy and reported satisfying results [19].

### Transfer in massive rotator cuff tears

#### Open surgery

Massive rotator cuff tears are described as tears of >5 cm or tears of two or more tendons [20, 21]. Treatment approaches and management of massive RC tears for shoulder surgeons are still controversial [20–24]. Chronic tears can cause retraction of the tendon and fatty atrophy of the muscle [25, 26]. Two or more tendon full-thickness tears can cause anterosuperior migration of the humeral head and thus arthropathy [27]. Latissimus dorsi tendon transfer is a good option in young patients with posterosuperior tears without arthritis where shoulder arthroplasty cannot be performed and the tear cannot be repaired [28, 29]. The main purpose of LD transfer in massive RC tears is to restore external rotation and vertical stability of the humeral head [28].

**Figure 3:**
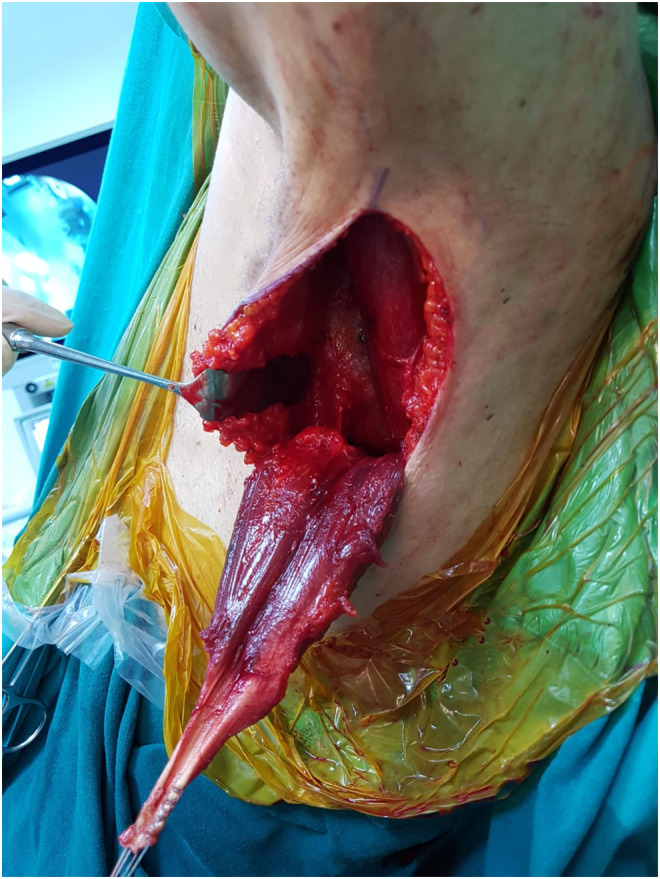
Preparation of the LD tendon by using axillary incision in a patient with massive rotator cuff tear.

**Figure 4:**
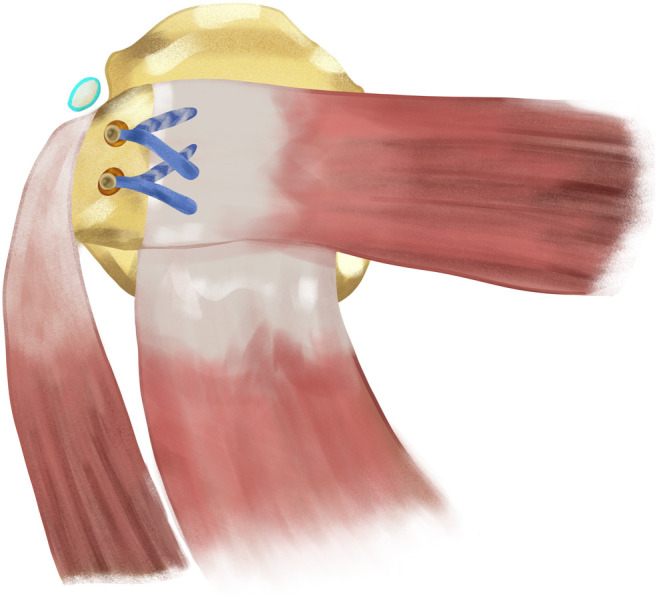
Axial scheme of the latissimus dorsi tendon transfer. The LD tendon is transferred to previously prepared footprint to restore external rotation and provide vertical stability of humerus in massive rotator cuff tear.

Christian Gerber described modern tendon transfer techniques in rotator cuff insufficiency based on the L’Episcopo technique [18]. The double incision was used in the technique described by Gerber for the first time in 1988 [13]. The first incision was performed in the axilla and the second incision was performed through the deltoid in the superior [13]. Successful results have been reported for this method applied by the authors in patients with an intact subscapularis tendon [30–32]. In the original technique, with a lateral decubitus position, the rotator cuff was identified by entering from the lateral of the acromioclavicular joint with the superior approach [33]. Following bursectomy, the size and shape of the tear were exposed, and the torn edges are debrided [33]. The RC was mobilized, and if it cannot be repaired, the LD transfer is considered [33]. Greater tuberosity is decorticated and an appropriate insertion area is prepared [34]. An axillary incision is made along the lateral edge of the LD [34]. The muscle is completely freed from the teres major and transected from the proximal humerus [34] (Figure 3). After exploration of the neurovascular structures, the tendon is transferred from the interval between the deltoid and infraspinatus-teres minor to the proximal humerus [34]. The LD tendon is reattached in the area prepared at the first incision (Figure 4) [35].

Although the muscle strength of teres major is three times higher than the latissimus dorsi, the teres major muscle is shorter and therefore must be fixed more anteriorly than necessary, curving between the teres minor and deltoid muscles while transferring is also one of teres major’s disadvantages [2]. Consequently, LD transfer has been the gold standard method by Gerber for years [2].

Over time, surgeons have turned to perform LD transfer with a single incision in massive RC tears to eliminate the handicaps caused by the double incision in the standard technique [36, 37]. Habermeyer described the LD tendon transfer technique using only the axillary incision to avoid deltoid injury caused by double incision [36]; Boileau, on the other hand, modified the original deltopectoral incision described by L’Episcopo and used the technique of transferring LD and teres major together [38].

In the single incision technique described by Habermeyer, the patient is placed in the lateral decubitus position [36]. A diagnostic arthroscopy is performed first, and the long head of the biceps is tenotomized if required [36]. After a triangular incision that is made from the axillary pouch to its apex and from the lateral border of the scapula to the inferior scapular angle, LD and teres major is detected. LD is freed from teres major [36]. The infraspinatus footprint is identified, and the tuberculum majus becomes prominent after the arm is brought to 90° of flexion and maximum external rotation [36]. After the infraspinatus insertion and preparation of the tuberculum majus area, LD is reattached further posteriorly and inferiorly with suture anchors compared to the standard technique [36]. This technique provides better functional results by avoiding complications of the second incision without damaging the deltoid [36].

Despite soft tissue releases, the LD tendon length can be insufficient to produce a good closure [39]. Augmenting the LD tendon with an allograft or xenograft patch may increase the muscle size and length [39]. Although some studies have indicated that augmentation has positive effects on the integrity of the tendon, there are also studies claiming the opposite [39, 40]. The authors do not routinely use allografts in a standard latissimus dorsi tendon transfer for irreparable posterosuperior rotator cuff tear [39, 40].

#### Arthroscopy assisted transfer

**Figure 5:**
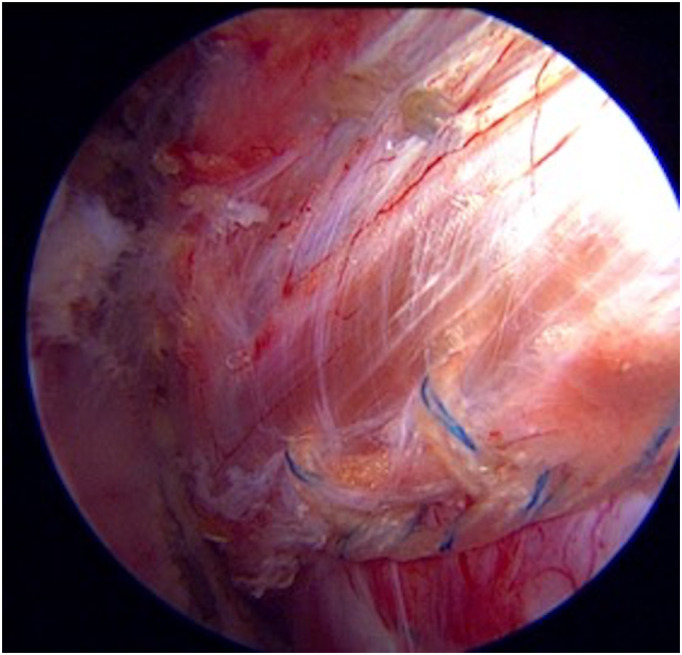
Arthroscopic view of transferred latissimus dorsi tendon. Right shoulder, view from lateral portal.

Another evolution of LD tendon transfer is the arthroscopic transfer technique in massive RC tears described by Gervasi [18]. In this technique, the shoulder joint is first visualized arthroscopically, and the tear is identified to confirm that it is not repairable and the presence of additional pathologies [18]. After the preparation of the footprint, the LD tendon is prepared for transfer using special retractors and large-diameter endoscopes with a 5–6 cm axillary incision [41]. This endoscopic method was first performed in 2003 and was published in 2007 [41]. Gas or water distension was not used during surgery when preparing the LD tendon endoscopically using the axillary approach [41]. The prepared LD tendon is moved to the subacromial area with the help of an arthroscopic grasper and sutures [41]. The LD is reattached near the intertubercular groove superior to the subscapularis tendon and at the insertion of the supra-infraspinatus tendons [41, 42] (Figure 5).

Later, further modifications of this method were described [43]. Using Doppler ultrasonography (USG), the location of the LD vascular pedicle was determined in the preoperative period, thus minimizing the size of the incision and increasing the accuracy of the incision site [44]. The necessity of avoiding the morbidity caused by the approaches applied during preparation, transfer, and fixation of the tendon in the deltoid muscle encouraged surgeons to use more minimally invasive techniques [44]. Therefore, all arthroscopic methods have been developed in LD tendon transfer surgery in irreparable RC tears [45, 46]. In all arthroscopic methods, the patient is placed in a lateral decubitus position using a device that allows full shoulder joint movements [16]. Axillary arthroscopic portals that allow visualization and working are used [45]. First, visualization is performed through the axillary portal located at the level of the LD anterior edge [45]. Teres major, latissimus dorsi muscles and thoracodorsal neurovascular pedicle are observed [45]. Tendons are carefully dissected, and sutures are placed on the lower and upper boundaries of the LD with a suture passer, which then helps to facilitate the mobilization of the muscle [45]. Later, arthroscopically, the LD tendon is transferred through the tunnel prepared between the deltoid and teres minor muscles and transferred to the supraspinatus footprint [45].

### Transfer in subscapularis tendon tears

Irreparable subscapularis tears are uncommon but difficult to manage when observed [47]. Subscapularis tears can cause posterosuperior instability, pain, and loss of internal rotation in the glenohumeral joint [33]. If there is a concomitant supraspinatus tear, then a vertical instability occurs, resulting in anterosuperior migration of the humeral head and loss of the anterior elevation of the shoulder joint [48]. Authors have described various reconstruction techniques for irreparable subscapularis tears [47]. Historically, the most commonly used technique described in the treatment of irreparable subscapularis tears is the transfer of the pectoralis major and minor [47–52]. However, the results obtained in the literature regarding these techniques are not satisfactory [47, 52, 53]. Although there was an improvement in pain scores in patients who underwent a pectoralis major transfer, the results obtained in internal rotation strength, active range of motion values, and stabilization of the humeral head were insufficient [47, 50, 53]. Consistent with the negative consequences of the transfer of the pectoralis major and minor muscles in irreparable subscapularis tears, Elhassan et al. studied the feasibility of the latissimus dorsi tendon transfer on cadavers in 2014 [54]. This reconstruction is possible, and there is no nerve entrapment after application [54]. In the surgical technique, the patient is placed in the beach chair position and a standard deltopectoral incision is used [54]. After the cephalic vein is protected, the tuberculum minus is identified and the remnant of the ruptured subscapularis tendon is exposed [54]. In chronic and/or recurrent subscapularis tears, the tendon can only be exposed under the coracoid, thus coracoid process osteotomy can be performed to provide better visibility [54]. Musculocutaneous and axillary nerves are exposed and protected [54]. The LD tendon is identified by descending towards the distal area of the deltopectoral interval [54]. After the LD tendon is released from the teres major, the LD tendon is removed from its insertion with a 5 mm bone piece using a saw or osteotome [54].

**Figure 6:**
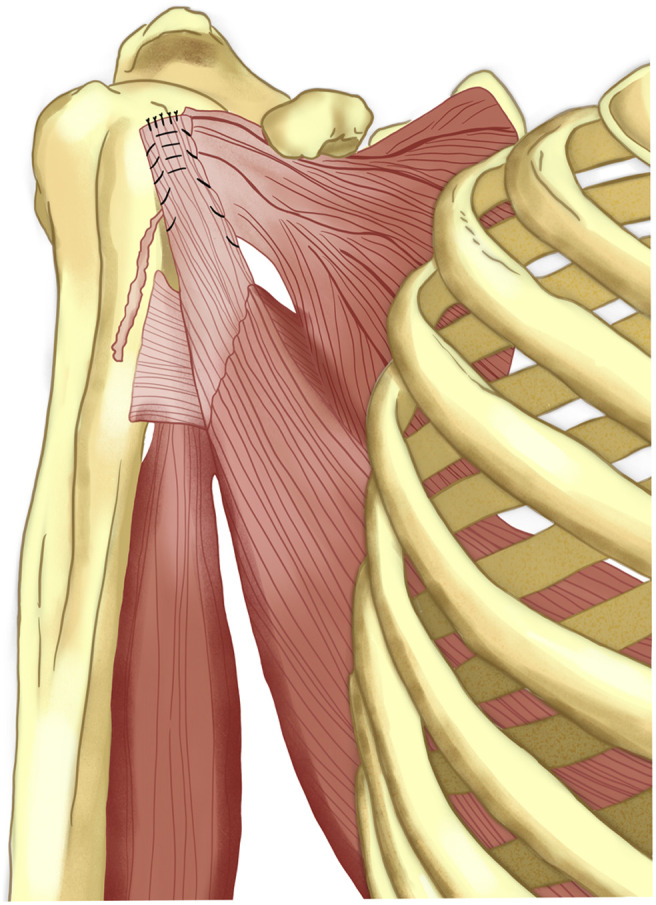
Latissimus dorsi tendon transfer for irreparable subscapularis tendon tear.

After preparing the attachment site of the subscapularis tendon on the tuberculum minus, the LD tendon is transferred (Figure 6).

When LD transfer was first described in irreparable subscapularis tears, studies were commonly cadaver studies and case reports. Later, case series began to be reported [55]. Accordingly, LD transfer was performed in irreparable subscapularis tears, and significant improvement was found in the range of motion and activity levels of patients [55]. Later, these open surgical techniques were modified and started to be performed with arthroscopy assistance [18].

### Transfer in reverse shoulder arthroplasty

Massive rotator cuff tears often result in superior migration of the humeral head in the long term [1]. This causes degeneration and loss of function in the glenohumeral joint [1]. Pseudoparalysis occurs in some patients as a result of an irreparable posterosuperior rotator cuff tear [25]. This situation causes a combined loss of forward elevation and external rotation (CLEER) [24]. Even isolated loss of external rotation (ILER) can be seen in some patients [24]. This defect in external rotation causes difficulties in people’s daily work [25]. In elderly individuals with loss of function and glenohumeral arthritis, reverse shoulder arthroplasty is a good option for restoring joint motion and increasing ROM [56]. In individuals with glenohumeral arthritis and massive posterosuperior rotator cuff tears, reverse shoulder arthroplasty can provide anterior elevation; however, the limitation of external rotation remains, resulting in negative patient satisfaction [57].

**Figure 7:**
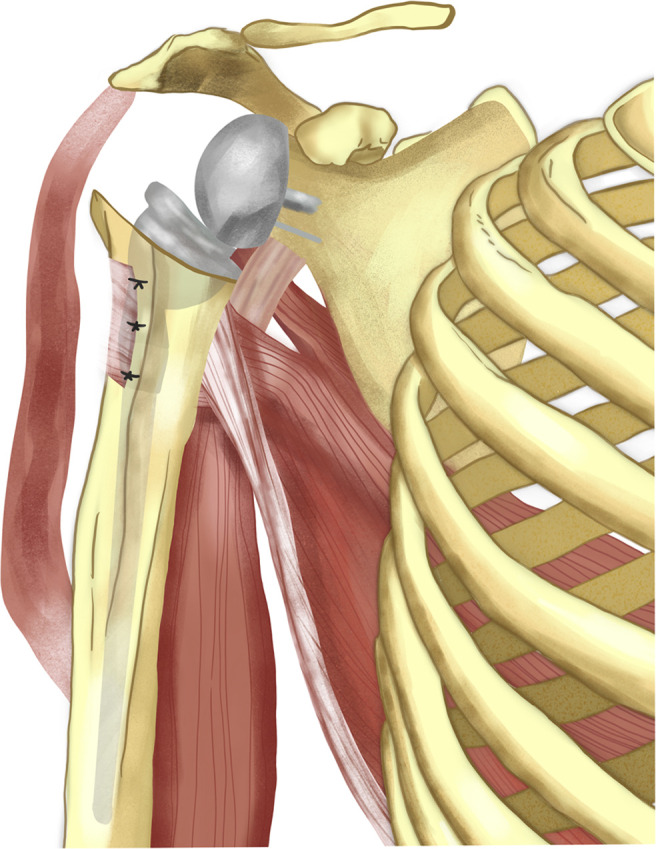
Latissimus dorsi tendon transfer in reverse shoulder arthroplasty.

Studies have shown that external rotation pseudoparalysis can be corrected by LD transfer, but this reconstruction does not contribute to elevation [58, 59]. To address the functional defect in patients with a combined loss of elevation and external rotation, Gerber et al. described LD transfer with reverse shoulder arthroplasty in 2007 [60]. In the same year, Boileau et al. described the transfer of LD and teres major tendons combined with a reverse shoulder arthroplasty [61]. This combined technique has been effective in painful pseudoparalytic irreparable posterosuperior rotator cuff tears with glenohumeral arthropathy, in the presence of functional deltoid muscle [60, 61]. In Gerber’s technique, a second anterior incision is added to the axillary incision used in classical LD transfer, while a single deltopectoral incision is used in the technique described by Boileau, which combines reverse shoulder arthroplasty with LD and teres major tendon transfer [4, 30]. After the patient is placed in the beach chair position, a single deltopectoral incision is made [61]. Neurovascular structures are preserved, and the insertions of teres major and LD muscles in the humerus are determined [61]. Teres major is generally located more posterior to the LD and attached to a wider base [62]. After the two tendons are sutured with different colors, they are removed from their insertions [61]. The purpose of the different sutures is to prevent the tendons from crossing each other after harvesting [61]. To avoid damage to the neurovascular structures, the tendons are dissected bluntly [61]. LD and teres major tendons are rotated around the humeral shaft and reattached to the pectoralis major tendon with Mason Allen stitches [61]. After, reverse shoulder replacement procedures are completed (Figure 7). In this context, LD transfer with reverse shoulder arthroplasty has provided a solution for indicated patients; LD transfer can restore external rotation and produce better functional outcomes [61, 63] (Table 1).

**Table 1:** Latissimus dorsi tendon transfer techniques.

Method	Aim	Surgical procedure	Advantages	Disadvantages
L’Episcopo [17]	To correct existing disability and restore muscle balance.	The contracted anterior tissues are released using Sever’s technique. Teres major and latissimus dorsi (LD) tendons are transferred to act as external rotators.	This technique does not only correct the deformity but also addresses the problem of muscle imbalance.	This is a salvage method.
Gerber et al. [13]	To close massive rotator cuff defect and exert an external rotation as well as act as a humeral head depressor.	Double incisions are performed. Harvesting the LD tendon with an axillary approach and transfer to treat the rotator cuff defects.	Symptomatic patients with irreparable cuff tears can be treated.	Neurological complications, deltoid avulsion, double incision
Habermeyer et al. [36]	To treat irreparable rotator cuff tears	A single incision is performed in the axillary pouch. LD tendon is detached from its insertion. The greater tuberosity is identified and LD tendon is transferred.	Single-incision	
			Less neurovascular complication	
Gerber et al. [60]	Combination of reverse shoulder arthroplasty with LD tendon transfer	LD tendon is harvested by axillary incision and joint replacement is made with deltopectoral or superolateral approach.	Restoration of both external rotation and elevation deficits	Double incision, longer surgery
Gervasi et al. [41]	Arthroscopic latissimus dorsi transfer for irreparable massive rotator cuff tears	The shoulder joint is first visualized arthroscopically. The prepared LD tendon is reattached at the footprint of supraspinatus tendon.	Arthroscopic assistance while reattaching tendon	Endoscopic graft harvesting is time-consuming and no benefits.
			Less wound complication	
			Avoiding deltoid harm	
Boileau et al. [38]	To treat irreparable rotator cuff tears as a part of reverse shoulder arthroplasty procedure.	An extended deltopectoral approach is used. LD and teres major tendons insertions are detached from the humeral periosteum. Two tendons are fixed directly to the pectoralis major stump.	Single-incision	Longer surgery
			Less neurovascular complication	
Elhassan et al. [54]	To address massive and irreparable subscapularis tendon tears	A standard deltopectoral approach is performed. LD tendon with bony attachment is harvested and transferred to the humeral head.	More satisfactory outcomes that pectoralis major/minor transfer	
			No nerve entrapment	
			Better anatomically and biomechanically	

## Conclusion

As a large, vascularized tendon that closes the rotator cuff defect, helps the deltoid muscle move more effectively, and can restore shoulder external rotation, the LD has been used in various ways due to these features. The starting point of LD transfer is to create a force against internal rotation contracture in obstetric palsy [16]. Besides, it has also been preferred as a musculocutaneous flap in plastic surgery [64]. LD flap was first described by Tansini and Watson et al. described the first free microvascular transfer of LD [64]. LD musculocutaneous flap is thought to be a useful option for hemipelvectomy, lower extremity, and breast reconstruction thanks to its neurovascular supply [64, 65]. Also, its large size and its ability to create external rotation suggested the use of this tendon in massive RC tears for defect closure and to increase functionality. Later, these techniques were advanced and started to be performed arthroscopically. The presence of subscapularis insufficiency in massive RC tears is contraindicated for LD transfer, but ironically, LD transfer has also been a good option for the treatment of subscapularis tendon tears in recent years. The addition of tendon transfer to arthroplasty surgery has led to good results in increasing the functionality of daily living activities in patients undergoing reverse shoulder arthroplasty. Various studies and surgical technique developments on the subject continue. This historical review guides surgeons who want to perform the described procedures.
